# Are Mendelian randomization investigations immune from bias due to reverse causation?

**DOI:** 10.1007/s10654-021-00726-8

**Published:** 2021-02-21

**Authors:** Stephen Burgess, Sonja A Swanson, Jeremy A Labrecque

**Affiliations:** 1 MRC Biostatistics Unit, University of Cambridge, Cambridge, UK; 2 Cardiovascular Epidemiology Unit, Department of Public Health and Primary Care, University of Cambridge, Cambridge, UK; 3 Department of Epidemiology, Erasmus MC, Rotterdam, Netherlands

Mendelian randomization uses genetic variants as instrumental variables to make causal inferences about the effect of a risk factor on an outcome [1, 2]. If a genetic variant satisfies the instrumental variable assumptions for the given risk factor and outcome [3], then an association between the genetic variant and the outcome implies the risk factor affects the outcome in some individuals at some point in the life-course [4]. Combining the instrumental variable assumptions with further assumptions and precise specification of the outcome (including specifying a time period for the outcome) allows valid testing of a more specific causal hypothesis and/or valid estimation of global or local, and point or period average causal effects [5].

Two motivations for Mendelian randomization are primarily stated: avoiding bias from unmeasured confounding and avoiding bias from reverse causation [6]. Reverse causation occurs when the outcome variable at an earlier timepoint, or a proximal precursor of the outcome (such as pre-clinical disease), has a causal effect on the risk factor which can bias estimates of the effect of the risk factor on the outcome. Though it can often be viewed as a specific form of confounding (when pre-clinical disease is a shared cause of the risk factor and outcome leading to violation of exchangeability conditions [7]), reverse causation has been treated as distinct from other forms of confounding in the motivation for Mendelian randomization [6, 8]. (We underscore that reverse causation does not imply that time flows backwards or somehow that future measurements influence the past, but that even if the outcome is measured at a later timepoint to the risk factor, either the outcome at an earlier timepoint or a precursor of the outcome may have influenced the measured value of the risk factor.)

An individual’s genetic code is fixed at conception. This implies that associations between genetic variants and subsequent outcomes are less vulnerable to bias from many sources of confounding and reverse causation. For example, environmental or lifestyle factors that occur post-conception cannot be a cause of the genetic variants and therefore cannot be a shared cause of the variants and outcome. Further protection from confounding comes from the random allocation of genetic variants during meiosis and from random mating within the population (although completely random mating is not plausible, mating is often plausibly random with respect to the genetic variants included in Mendelian randomization analyses) – meaning that genetic variants are often independent of confounding factors other than ancestry [9, 10].

It has also often been stated that the fixed nature of the genetic code provides complete immunity to bias from reverse causation in Mendelian randomization studies because genetic variants must precede the outcome in time. For example, Davey Smith and Ebrahim [8] wrote about “the lack of possibility of reverse causation as an influence on exposure–outcome associations in both Mendelian randomization and randomized controlled trial settings” and remarked “the instrument will not be influenced by the development of the outcome (i.e., there will be no reverse causation)”. Here, we demonstrate how reverse causation can lead to bias in Mendelian randomization analyses. For each scenario, we show that even though the variant–outcome associations may not suffer from reverse causation, reverse causation between the risk factor and outcome either in individuals or across generations can result in bias in Mendelian randomization analyses. That is, even though the outcome may not cause the genetic variant (and thus the variant–outcome association may not seem to suffer from reverse causation), the type of reverse causation that affects traditional analyses may still indeed bias estimates from Mendelian randomization studies (when a Mendelian randomization analysis is undertaken to estimate a causal parameter) and invalidate causal conclusions (when a Mendelian randomization analysis is undertaken to test a causal hypothesis) [11, 12]. In the former case, bias relates to a specified average causal effect estimate; in the latter case, bias relates to the test statistic for a causal hypothesis.

## Scenario 1. Genetic association with the risk factor is not primary

**Fig. 1:**
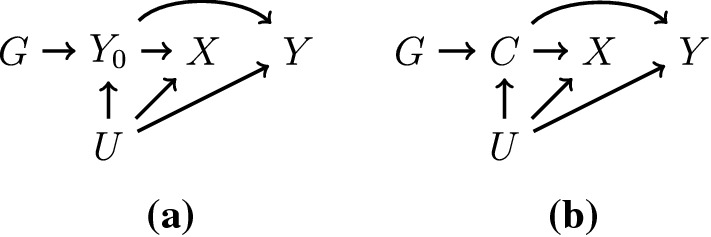
Diagrams illustrating relationships between a genetic variant (*G*), risk factor (*X*), and an outcome (*Y*), where the effect of the genetic variant on the risk factor is a) through its effect on the outcome previous to the risk factor ($Y_0$) and b) through a confounder (*C*) – a common cause of risk factor and outcome. Unmeasured confounding is represented by *U*. In both diagrams, the effect of interest is the effect of *X* on *Y*

The first mechanism we consider is that a genetic variant is associated with the risk factor via a primary effect of the variant on the outcome variable or on a precursor of the outcome (Fig. 1). By primary, we mean that the risk factor occurs upstream of the outcome in all directed causal paths from the genetic variant to the outcome; that is, any directed causal pathway from the genetic variant to the outcome at a specified follow-up time pass via the risk factor at preceding times. In the opposite scenario, the genetic association with the risk factor is not primary if the effect of the genetic variant on the risk factor is mediated (at least in part) by the outcome.

As an example, testosterone has been hypothesized as a possible causal risk factor for polycystic ovary syndrome (PCOS). Genetic variants that predict testosterone concentration in women have been shown to be associated with risk of PCOS [13]. However, one of the symptoms of PCOS is increased testosterone. Therefore, it may not be that elevated testosterone that leads to increased risk of PCOS, but increased predisposition to PCOS that leads to elevated testosterone levels. Genetic variants identified as instruments for testosterone may not affect testosterone directly, but rather via their association with PCOS. The variants may affect risk of PCOS directly (Fig. 1a) or indirectly via an alternative risk factor for PCOS or pre-clinical PCOS (Fig. 1b). The genetic variants are still primary in the causal chain, but reverse causation between the putative risk factor and outcome means that the variants influence the risk factor secondarily. In this case, an association between the genetic variants and outcome can be present without a causal effect of the risk factor on the outcome.

As a further example, genetic variants associated with aspirin treatment were used in a Mendelian randomization analysis to assess the effect of aspirin use on risk of lung cancer [14]. However, all the genetic predictors of aspirin use are all also associated with risk of coronary heart disease [15]. It is likely that the genetic associations with aspirin use arise due to individuals with coronary heart disease or high levels of risk factors for coronary heart disease being preferentially prescribed aspirin. As coronary heart disease and lung cancer are competing outcomes, the reported protective effect of aspirin on lung cancer risk in the Mendelian randomization analysis may be due to the genetic associations with aspirin being secondary to their effects acting via coronary heart disease and/or risk factors for coronary heart disease. This could lead to alternative pathways from the genetic variants to the outcome not via the risk factor.

Genetic associations will broadly be lesser in strength when the path from the gene to the trait is less direct. However, as sample sizes for genetic discovery increase, it is increasingly likely that some genetic associations with risk factors are secondary to their association with another variable. The chances of finding such a variant also increase when reverse causation between the risk factor and outcome is stronger. In other words, if Mendelian randomization is being used specifically because of concerns about reverse causation in a traditional observational analysis, the risk of bias due to reverse causation via this mechanism in Mendelian randomization will also be higher. In this scenario, not only are effect estimates expected to be biased, but tests of causal null hypotheses are also not valid.

## Scenario 2. Feedback mechanism

**Fig. 2:**
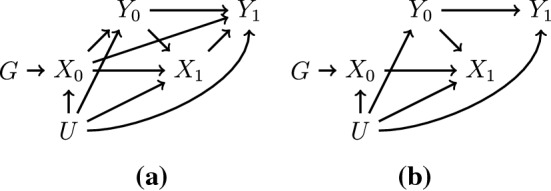
Diagrams illustrating time-varying relationships between a genetic variant (*G*), risk factor (*X*), an outcome (*Y*) and unmeasured confounder (*U*) at time 0 and time 1 (indicated by subscripts) where **a** the genetic variant has a primary effect on the risk factor, and there are bidirectional effects between the risk factor and outcome and **b** the genetic variant has a primary effect on the risk factor, but only the reverse causal effect of the outcome on the risk factor is present, meaning that genetic variant is independent of the outcome. The effect of interest is the joint effect of $X_0$ and $X_1$ on $Y_1$

Secondly, Mendelian randomization studies with genetic variants that have direct effects only on the risk factor (i.e. they do not directly affect the outcome) can still suffer from bias due to reverse causation. For instance, if the risk factor influences the outcome and the outcome influences the risk factor at a later time-point (Figure 2a), then genetic associations with the risk factor will be distorted, and Mendelian randomization estimates may be misleading.

As an example, genetic variants that predict obesity have been shown to associate with income in women [16]. However, income affects many lifestyle factors, including obesity, leading to a feedback loop. A similar story can be told for cigarette smoking and obesity: genetic predictors of obesity associate with increased smoking prevalence (perhaps smokers seeking to reduce weight) [17], but genetic predictors of cigarette smoking associate with decreased weight (as cigarette smoking is an appetite suppressant) [18]. Depending on the strength and direction of the reverse causal effect and the prevalence of the outcome, genetic associations with the measured value of the risk factor can be over- or under-estimated due to reverse causation [19]. However, some tests of causation will be valid regardless of the presence of this type of reverse causation [5]. For instance, this type of reverse causation will not affect the validity of a test of the sharp causal null (that there is a causal effect in at least one person at one point in time) of the risk factor on the outcome assuming the instrumental variable assumptions hold (Fig. 2b). This is because an association between the genetic variant and outcome still reflects the existence of pathways that go through the risk factor first, even though effect estimation cannot as readily tease apart the feedback loops.

Feedback scenarios can occur other than due to reverse causation. A different feedback scenario is that individuals with high levels of a risk factor will preferentially take medication to lower the risk factor. For example, individuals with high levels of cholesterol are more likely to take cholesterol-lowering medication, and similarly for blood pressure. The reverse is true for factors that are beneficial for health outcomes. For example, pregnant women with low iron status are more likely to take iron supplements [20]. In extreme cases where intervention on the risk factor is common and substantial, it may even be that medication or supplementation attenuates completely or even reverses genetic associations with the risk factor. This is particularly important in the example of iron and pregnancy, as the risk factor of interest is not maternal iron levels in general, but maternal iron levels during the critical period of pregnancy.

## Scenario 3. Cross-generational effects

**Fig. 3:**
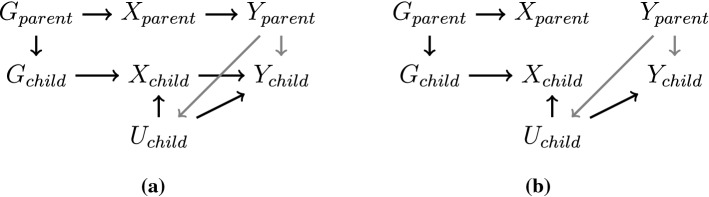
A cross-generational diagram of genetic variant (*G*), risk factor (*X*), and outcome (*Y*) in both the parent and child. The potential cross-generational reverse causal effect of parental outcome on offspring confounder or outcome is displayed in grey. If estimating the effect of the risk factor in the child on the outcome in the child, when the risk factor causes the outcome in either or both generations (panel a), Mendelian randomization estimates will typically be non-null, but biased. When the risk factor does not cause the outcome in either generation (panel b), Mendelian randomization estimates will not be biased and will provide a valid test of the sharp causal null hypothesis. Shared causes of the parent’s exposure and outcome, and their effects on the child’s exposure and outcome that are not relevant to the bias under study, are omitted for clarity

Finally, even though they are fixed at the start of an individual’s life, genetic variants are inherited from an individual’s parents. Hence when considering effects that may span across generations, an individual’s genetic variants are no longer primary in the causal chain. Therefore, when trying to estimate the effect of a risk factor for individuals in one generation, the outcome in the parental generation could influence the outcome or confounders of the outcome in the target generation directly, leading to a pathway from the genetic variants of an individual in the target generation to their outcome that is not via their risk factor (Fig. 3). While this scenario stretches the common understanding of reverse causation somewhat, this is still an example of the outcome influencing a downstream variable, even if the outcome in this case is in the previous generation, and so we believe it is worth discussing while addressing the topic of reverse causation.

For instance, the same genetic variants that predispose an individual to increased alcohol consumption also predisposed at least one of the individual’s parents to increased alcohol consumption. Outcomes in the offspring generation may be driven by the outcomes caused by the parents’ alcohol consumption, rather than from the offspring’s alcohol consumption directly. Hence there may be causal effects of alcohol even amongst individuals who themselves do not drink. Additionally, increased parental predisposition to drinking alcohol may affect offspring alcohol consumption, distorting Mendelian randomization estimates. As a further example, genetic variants associated with body mass index may be associated with outcomes not only due to the effect of obesity in the individuals observed, but also due to obesity and its consequences in the parent generation.

From the perspective of aetiology, this is not always such a serious problem as even if the offspring outcomes are driven by the risk factor and its consequences in the parents, it is still the risk factor that is causal for the outcome. However, from the perspective of intervention, changing the risk factor in the offspring may not lead to the consequences for offspring outcomes that are predicted by straightforward interpretation of a Mendelian randomization estimate. Hence Mendelian randomization investigations with cross-generational effects are able to assess the causal relevance of the risk factor in a broad sense, in that they can test the sharp causal null that the risk factor affects the outcome in at least one generation. However, the pathway by which the risk factor influences the outcome may be driven by the effect of the risk factor in a previous generation.

## Discussion and conclusion

In this short manuscript, we have discussed three ways in which Mendelian randomization analyses may be susceptible to bias due to reverse causation. Although in some cases a causal hypothesis can still be validly tested, in other cases causal inferences of all types from the approach may be unreliable. Several methodological researchers have already cautioned against interpretation of causal effect estimates from Mendelian randomization as the expected impact of intervening on the risk factor in a clinical setting, or even advised against presenting causal effect estimates at all [4, 11, 21]. This manuscript provides further reasons for caution not only in the interpretation of effect estimates, but also in the validity of causal null hypothesis testing. It is important to appreciate context when interpreting findings from a Mendelian randomization analysis, and to be aware that the estimated causal effect of the risk factor (which typically gets interpreted as the impact of a lifelong change in the trajectory of a risk factor) may not be achievable by a practical intervention on the risk factor in the target population. Drawing directed acyclic graphs, carefully defining the risk factor and outcome (in a way that acknowledges time), and thinking closely about how the genetic variant influences the trajectory of the risk factor will help analysts to precisely define the causal effect of interest, and hence detect the possibility for findings to be influenced by reverse causation.

There are several approaches that can be taken by investigators to mitigate or identify bias due to reverse causation. Some of this guidance follows best practices for Mendelian randomization studies more broadly [12]. Overall, where possible, Mendelian randomization analyses should be performed using genetic variants for which the mechanism of association of the variants with the risk factor is both primary and well-understood. As a consequence of this, investigators should prioritize Mendelian randomization analyses for risk factors that have proximal genetic variants. When the mechanism linking genetic variants and risk factors is unclear or distant, inferences from Mendelian randomization generally carry less evidential weight. As for more advice more specific to the scenarios considered here, first, statistical methods have been developed to help distinguish whether genetic variants primarily influence the risk factor or another variable (as per Scenario 1). The MR-Steiger method measures the proportion of variance explained by a genetic variant in the risk factor and in the outcome [22] and can be used to flag for removal from the analysis variants that are more strongly linked to the outcome than the risk factor. This method is not guaranteed to identify Scenario 1, and is sensitive to measurement error. Secondly, simulations can be used to explore the extent of bias due to feedback mechanisms (as per Scenario 2), although this relies on strong assumptions about the temporal nature and magnitude of the feedback [19]. Thirdly, statistical methods have been developed to consider cross-generational effects (as per Scenario 3) when data are available on parents and offspring [23, 24]. If such data are not available, researchers should express caution in the interpretation of a Mendelian randomization investigation when it is plausible that causal effects may span across generations. Scenarios 2 and 3 further underscore the general recommendation to view Mendelian randomization as primarily testing a causal null hypothesis rather than estimating a causal effect [12].

In conclusion, while it is fair to say that Mendelian randomization investigations offer some protection against biases that can be conceptualized as reverse causation, it is not reasonable to claim that Mendelian randomization investigations are totally immune from the phenomenon. Researchers should consider carefully whether their findings could be explained by genetic variants having a primary association with the outcome, and how previous versions of an outcome (within an individual or across generations) can impact the stated risk factor.
